# The comfort of approach: self-soothing effects of behavioral approach in response to meaning violations

**DOI:** 10.3389/fpsyg.2014.01568

**Published:** 2015-01-09

**Authors:** Willem W. A. Sleegers, Travis Proulx

**Affiliations:** Department of Social Psychology, Tilburg UniversityTilburg, Netherlands

**Keywords:** meaning violation, threat, approach motivation, avoidance motivation, BIS/BAS

## Abstract

People maintain systems of beliefs that provide them with a sense of belongingness, control, identity, and meaning, more generally. Recent research shows that when these beliefs are threatened a syndrome of negatively valenced arousal is evoked that motivates people to seek comfort in their ideologies or other personally valued beliefs. In this paper we will provide an overview of this process and discuss areas for future research. Beginning with the neural foundations of meaning violations, we review findings that show the anterior cingulate cortex is responsible for detecting inconsistencies, and importantly, that this is experienced as aversive. Next, we evaluate the evidential support for a psychophysiological arousal response as measured by cardiography and skin conductance. We discuss how current theorizing proposes that subsequent behavioral approach ameliorates the negative arousal and serves as an effective, well-adapted coping response, but we also aim to further integrate this process in the existing threat-compensation literature. Finally, we speculate on whether approach motivation is likely to result when one feels capable of handling the threat, thereby incorporating the biopsychosocial model that distinguishes between challenge and threat into the motivational threat-response literature. We believe the current literature on threat and meaning has much to offer and we aim to provide new incentives for further development.

## INTRODUCTION

Over the course of the last half a century, research on coping has identified a plurality of ways that people deal with stress (Zimmer-Gembeck and Skinner, 2010). To illustrate, people commonly find comfort in actions such as seeking out social contacts, engaging in wishful thinking, eating comforting foods and taking hot showers. As we will argue in this review, people will also approach and affirm committed values, ideals, ideologies, and worldviews. Generally, comfort is sought in response to threatening experiences, and we believe that the threat-compensation literature has much to offer on the topic of self-comforting strategies. In this literature an integrative picture is emerging that states motivational processes underlie the response to a certain class of stressors we describe as *meaning violations* (e.g., McGregor et al., 2010; Proulx et al., 2012). It is argued that when faced with a meaning violation, people show an initial defensive reaction marked by anxiety, vigilance, and avoidance, which subsequently switches to a motivational state of behavioral approach that ameliorates this anxiety, thereby serving a palliative, self-comforting function. In this review, we will provide an overview of the neuroaffective and psychophysiological processes that have been linked to the typical compensation behavior of the threat-compensation literature, and suggest directions for future research in this field.

## DEFINING MEANING VIOLATIONS

The threat-compensation literature is filled with psychological theories aimed at describing and understanding people’s reactions to particular types of threat (Proulx, 2012). Of these threats, traumatic experiences (e.g., sexual abuse, natural disasters, violent attacks) undoubtedly rank among the most impactful. These experiences threaten core motivations such as our desire to avoid death and attain personal control—two motives that have received much attention in the social psychological literature, framed in terms of prominent perspectives such as terror management theory (Burke et al., 2010) and compensatory control theory (Kay et al., 2009). Traumatic experiences, however, do not simply create a single dose of proximal anxiety. In addition to the clear physical hazards they often represent, they also impact the way in which we understand ourselves and our world. Instead of living in a safe and just world—a common assumption—they force us to realize we live in a world of danger and injustice. This implication initiates a second “dose” of anxiety (Janoff-Bulman, 1992), whereby the threat to physical safety is compounded by shattered assumptions. Although the context of a traumatic experience easily evokes the understanding that related cognitions are important for well-being, Bruner and Postman (1949) used a relatively trivial perceptual anomaly to reach similar conclusions. They presented people with reverse colored playing cards (e.g., a black two of hearts) an experience that did not match their expectations, which elicited signs of personal distress.

Cognitive dissonance theory has formally described this mismatch between beliefs and experiences along with the aversive feeling of dissonance that results (Festinger, 1957, or see Brehm, 2007). Subsequent theorists have developed this focus on cognitive consistency and uncertainty. For example, lay epistemic theory (Kruglanski et al., 2010), self-verification theory (Swann and Read, 1981), and uncertainty management theories (e.g., Uncertainty Reduction, Hogg, 2007; Uncertainty Management, van den Bos, 2001) all focus on a motivation to replace dissonant cognitions with consonant cognitions and perceived clarity. One way to achieve this is by assimilating experiences so that they are consistent with one’s expectations. Bruner and Postman (1949) found that people often reported not seeing a black two of hearts, but actually an expectancy-congruent black two of spades. Alternatively, they could have accommodated their understanding by realizing they were perceiving an altered deck of playing cards. This form of dissonance reduction was commonly reported in classic cognitive dissonance paradigms where participants—mostly students—were induced to behave in ways that contradicted their attitudes (e.g., argue in favor of a tuition increase). Subsequent accommodation of the dissonant behavior took place in the form of a change in attitude toward the tuition fee, thereby resolving the dissonance. In sum, assimilation, and accommodation can be seen as compensatory responses to resolve inconsistencies in cognitions.

Psychologists have furthermore observed that in addition to assimilation and accommodation, people can show a heightened commitment to alternative beliefs or values following many of the same inconsistencies that elicit assimilation or accommodation behaviors. For example, arguing for a tuition increase results in a change in attitude toward the tuition fee, but not if participants are first given the opportunity to affirm of unrelated values such as political beliefs (Steele and Liu, 1983). Hundreds of subsequent studies have shown active affirmation of values following reminders of mortality (Burke et al., 2010), lack of control (e.g., Kay et al., 2010), and the experience of uncertainty (e.g., van den Bos et al., 2006).

The abundance of threat-related theories almost invariably led to the development of more integrative perspectives. According to the meaning maintenance model (MMM; Heine et al., 2006; Proulx and Inzlicht, 2012), any inconsistency between experience and expectation evokes a syndrome of negative arousal that motivates compensation efforts. According to the reactive approach motivation model (RAM; McGregor et al., 2010), threats represent cues to goal conflicts that cause anxious uncertainty that serves an approach motivation function. More generally, these integrative models all frame threat-compensation effects in terms of discrepancies between perceptions, beliefs, or conflicting motivations. We see these discrepancies as affecting meaning, or the expected relationships that allow us to make sense of our experiences. To distinguish between threats that stem from negatively self-relevant situations (e.g., a dangerous predator, a robber) and sources of inconsistency [e.g., paradigm violations (Bruner and Postman, 1949), prediction errors (Hajcak and Foti, 2008)] that affect psychological motivation, we refer to the latter as meaning violations. While meaning violations may also have negatively self-relevant implications [e.g., worldview-violating personal tragedies (Janoff-Bulman, 1992)], the presence of inconsistency may be both necessary and sufficient to evoke the state of uncertainty that underlies the common aversive reactions, whether they follow from existential reminders, lack of control, behavioral dissonance, epistemic uncertainty or goal conflicts. This is followed by a compensatory reaction that resolves the aversive uncertainty caused by the meaning violation.

## THE PHYSIOLOGY OF MEANING VIOLATIONS

### BEHAVIORAL APPROACH AND FRONTAL ASYMMETRY

Gray (1982) published “The Neuropsychology of Anxiety” (since updated; Gray and McNaughton, 2003) that describes anxiety as activity of the behavioral inhibition system (BIS). A threat, however, generated, activates the BIS and produces behavioral inhibition, heightened arousal, and increased vigilance. As a result, ongoing behavior is halted and the environment is scanned for further threatening cues. In contrast to the behavioral inhibition system, a second system is responsible for reengaging behavior, known as the behavioral approach system (BAS; also known as the behavioral activation system). The BAS responds to reward cues, non-punishment and escape from punishment. This state is marked by attentional narrowing and feelings of hope, elation, and happiness.

Gray’s model of anxiety is mainly a neuropsychological model and, and while it is based in large part based on animal models, several human neurophysiological substrates have been proposed to underlie the BAS and BIS. Some of these substrates are now being investigated in the context of meaning violations. These involve the frontal areas of the brain, potentially the lateral and orbital regions of the prefrontal cortex. This is based on studies showing asymmetrical activation in frontal areas during approach and avoidance motivations (see, Davidson, 1992; Coan and Allen, 2003). Various psychological states elicit a frontal asymmetry that is consistent with a BAS state interpretation. For instance, Sutton and Davidson (1997) measured prefrontal asymmetry using EEG and linked this to self-report measures of BIS and BAS, using the BIS/BAS scale developed by Carver and White (1994).

The BAS scale assesses people’s tendency to experience positive affect and behavioral activation in goal-oriented situations. The BIS scale assesses the tendency to experience negative affect and behavioral inhibition in the face of threats. Sutton and Davidson (1997) found that greater left prefrontal activation was correlated with higher levels of BAS strength, whereas those with greater relative right prefrontal activity reported greater BIS strength. They also ruled out alternative explanations such as positive and negative affect confounds that are associated with BAS and BIS, respectively. These findings have also been shown in a study by Harmon-Jones and Allen (1997), who linked frontal cortical activity to self-report measures of BIS and BAS. Pizzagalli et al. (2005) performed a source localization study to gain more insight into the underlying structures responsible for the asymmetry. They found a correlation between activity in the dorsolateral prefrontal and medial orbitofrontal regions and a bias for reward-related cues [also see Berkman and Lieberman (2010)]. This further supports not only the relationship between frontal asymmetry and BAS, but also provides some insight into the anatomical details of this relationship.

At first, however, it was believed that frontal asymmetry was related to emotional valence, with greater left frontal asymmetry being linked to positive affective processing styles and vice versa (Fox, 1991; Jones and Fox, 1992; Wheeler et al., 1993). Yet, the previously discussed studies show the functioning is less related to emotional valence, and actually favor a motivational orientation interpretation. One particular study by Berkman and Lieberman (2010) has demonstrated that prefrontal asymmetry is associated with action motivation and not with stimulus valence. In their study, they compared approach/avoidance actions vs. stimulus valence using a novel goal pursuit task. Functional magnetic resonance imaging (fMRI) revealed an increased left activation in the dorsolateral prefrontal cortex during approach (vs. avoidance) actions irrespective of the valence of the stimulus. No such asymmetry was observed for pleasant compared to unpleasant stimuli. Additionally, individual differences in approach–avoidance motivation moderated the effect such that increasing trait approach motivation was associated with greater left-sided asymmetry during approach actions.

This interpretation, that frontal asymmetry reflects BAS, is further bolstered by studies linking frontal asymmetry to psychological constructs related to BAS motivation, such as depression and anger. Depression is argued to consist partially of a lack of motivation to approach. Consistently, depression has been linked to lower levels of relative left frontal activity (Henriques and Davidson, 1990; Allen et al., 1993). Anger, despite having a negative affective valence, has also been linked to greater left frontal activity (Harmon-Jones and Allen, 1998; Harmon-Jones, 2003). The link between anger and frontal asymmetry has also been supported through means of transcranial magnetic stimulation; which has shown that decreasing activity in the left prefrontal cortex lowers a memory bias for angry faces (van Honk and Schutter, 2006). Frontal asymmetry has also been shown in people who are in a promotion-oriented state (i.e., focused on gaining reward instead of avoiding losses), as opposed to an avoidance orientated state (Amodio et al., 2004). Finally, affecting frontal asymmetry through biofeedback techniques has been shown to increase self-reported affect and facial muscle activity in response to emotionally evocative film clips (Allen et al., 2001). These findings thus support the interpretation that frontal asymmetry is related to behavioral activation.

### BEHAVIORAL INHIBITION AND THE ANTERIOR CINGULATE CORTEX

Although many studies show a link between frontal asymmetry and behavioral activation-related outcome measures, the link between frontal asymmetry and behavioral inhibition is not always shown (Coan and Allen, 2003). Often studies lack the potential for greater insight into to the anatomical functioning of the underlying structures (Davidson, 2004), mostly due to the fact that non-spatial sensitive measures such as EEG are being used (but see Berkman and Lieberman, 2010 for an exception). EEG studies have, however, found other potential markers for BIS activation, and these markers have also been linked to meaning violations. These markers suggest the involvement of the anterior cingulate cortex (ACC). The ACC receives input from the limbic lobe, including the orbitofrontal cortex and the amygdala, as well as other nociceptive sources. For this reason it has been argued that the ACC serves a critical function for emotional and motivational factors (Pandya et al., 1981; Van Hoesen et al., 1993; Vogt et al., 1993; Morecraft and Van Hoesen, 1998; Bush et al., 2000).

The exact function of the ACC is still controversial. Research on error related negativity (ERN) suggests various possibilities. The ERN is a negative voltage deflection measured over the fronto-central scalp that appears to reflect activation of the ACC (Dehaene et al., 1994; Miltner et al., 1997). The ERN is elicited when people commit errors, or specifically, when they receive feedback about having committed an error, and usually appears between 50 and 100 ms after the feedback (Falkenstein et al., 1990; Gehring et al., 1993; Nieuwenhuis et al., 2004). Various models of the function of ERNs exist and they suggest that the ERN reflects either a conflict monitoring function (Botvinick et al., 2001; Yeung et al., 2004) or an evaluative function based on expectations developed during learning history (reinforcement-learning theory; Holroyd and Coles, 2002). In the latter construal, the ERN is an indication that events are worse than anticipated, or better than expected. Luu et al. (2000) have proposed that the ERN may signify affective processing in response to errors. This proposal is based on evidence that the magnitude of the ERN is affected by motivational and affective variables. Individuals with symptoms of depression (Chiu and Deldin, 2007), obsessive–compulsive disorder (Gehring et al., 2000; Hajcak and Simons, 2002; Hajcak et al., 2008), and generalized anxiety (Hajcak et al., 2003, 2004) show greater ERNs. Additionally, ERN activity has been associated with stronger skin conductance responses (Hajcak et al., 2004) and a more pronounced startle response following threat (Hajcak and Foti, 2008), while removal of this brain structure is associated with flat affect and a lack of distress (Corkin et al., 1979; Critchley et al., 2003). Similar to previously mentioned studying linking self-reported BAS to frontal asymmetry, Amodio et al. (2008) have linked self-reported BIS to ACC functioning. They found that self-reported BIS was uniquely related to the ERN in a Go/No-Go task, but not self-reported BAS. Moreover, BIS was also related to the N2, a negative potential that peaks about 250 ms after the onset of a No-Go trial; and is believed to arise similarly from the ACC (van Veen and Carter, 2002; Nieuwenhuis et al., 2003). These findings, and those discussed earlier, point toward the ACC being a crucial component of the BIS.

One of the most interesting findings in the threat-compensation literature has been that the ACC responds similarly to what we describe as meaning violations. For example, Quirin et al. (2012) showed that by letting participants answer questions about their fear of death, increased ACC activation could be observed (as well as activation in the amygdala and the caudate nucleus). The ACC activated relative to answering questions about dental pain, indicating this effect can go beyond that of negatively self-relevant events. ACC activation has also been demonstrated in response to the experience of cognitive dissonance. For example, Kitayama et al. (2013) asked participants to make decisions regarding CDs that differed in attractiveness, sometimes facing an easy choice (between two CDs that differ greatly in attractiveness, i.e., no cognitive dissonance) and a sometimes difficult choice (between two CDs that are similar in attractiveness, i.e., cognitive dissonance). They found that the cognitive dissonance eliciting choices resulted in activation of the dorsal ACC. Additionally, they found that these choices also resulted in activation of areas related to emotional distress (left anterior insula). Furthermore, they could predict a change in attitude toward the CDs that resolves the cognitive dissonance with activity in the posterior cingulate cortex. van Veen et al. (2009) used a similar setup to also predict attitude change based on neural activity in the cingulate cortex. They scanned participants with fMRI while they argued that the scanner environment—an uncomfortable environment—was, in fact, comfortable. Activity in the dorsal ACC, as well as activity in the anterior insula, predicted their change in attitude. These findings point toward a role of the ACC in resolving cognitive dissonance.

Additional studies have linked the ACC to meaning violations. For example, Salomons et al. (2004) manipulated the controllability over a painful stimulus and found that having less control was associated with increased ACC activity. Goal uncertainty has also been found to affect the ACC (Tullett et al., 2013), and a line of research has revealed that the ACC also plays a prominent role in how people respond to experiences of social isolation. In this line of research, participants play a ball tossing game (ostensibly) with other participants, who at a certain point stop throwing balls to the participant, or do so with such a low frequency that the participant experiences a lack of social inclusion. These studies consistently show cues of ostracism (not receiving the ball) evoke activity in the ACC (Eisenberger et al., 2003; Masten et al., 2009; Bolling et al., 2011, 2012; Moor et al., 2012). Some argue that part of the role of the ACC is due to the unexpected nature of not receiving a ball, and thus point to violation of expectations (e.g., Bolling et al., 2011). Indeed, expectancy violation as been argued to be the root cause of the aversiveness that follows from meaning violations (Proulx and Inzlicht, 2012) and is related to ACC activity (Oliveira et al., 2007).

### BEHAVIORAL INHIBITION AND CARDIOVASCULAR THREAT RESPONSE

Physiological indications of meaning violations are not limited to neural responses. The biopsychosocial model (BPSM) of arousal regulation (Blascovich and Tomaka, 1996; Blascovich, 2008) defines specific patterns of cardiovascular responses to threats. Specifically, the model states that when an individual faces a threat (i.e., negative appraisal of the situation) a malignant pattern of increasing cardiac or myocardial performance should occur, accompanied by stable or increasing vascular resistance caused by activation of the pituitary-adrenal-cortical (PAC) axis. PAC activity is thought to be under the control of the brain centers previously discussed as BIS (Gray and McNaughton, 2003).

Substantial evidence has accumulated supporting the contention that meaning violations also produce marked changes in sympathetic nervous activity. As early as the late 1960s, it has been shown that participants forced to choose between similar alternatives—and therefore experience cognitive dissonance—show greater decreases in finger pulse amplitude (Gerard, 1967), an index of a physiological readiness response as blood flows away from the periphery of the body. As well, studies showing that performing attitude-discrepant behaviors also leads to an increased galvanic skin response (GSR; Croyle and Cooper, 1983; Elkin and Leippe, 1986; Harmon-Jones et al., 1996). Losch and Cacioppo (1990) have offered additional evidence that cognitive dissonance increases arousal as measured by GSR, and have further shown that subsequent attitude change only occurs when people experience this arousal as explicitly unpleasant.

Other meaning violations, produce similar modes of arousal. For example, uncertainty about interacting with outgroup members has revealed patterns of cardiovascular reactivity consistent with threat (Blascovich et al., 2001), and so too has the case of uncertainty produced by the possibility of experiencing an electric shock (Monat et al., 1972). Similarly, cardiovascular responses indicating aversive arousal have been observed in participants interacting with partners that violate expectancies (Mendes et al., 2002, 2007), social threat (Hawkley et al., 2011; Van Beest and Scheepers, 2013) and a combination of these dimensions: unexpected social rejection (Moor et al., 2010).

## APPROACH AS A PALLIATIVE

After the initial aversive response to a meaning violation, people show an array of compensatory behaviors. Often, these are direct attempts to resolve the source of the violation. For example, people excluded from social interaction increase their interest in interaction with other people—strangers included (Maner et al., 2007)—and they try to fit in with others more by increasing their compliance (Williams, 2007, 2009; Carter-Sowell et al., 2008). Or, in the case of behavioral dissonance, students who are asked to argue in favor of a tuition increase will subsequently change their attitudes to resolve this attitudinally inconsistent behavior (Harmon-Jones et al., 2008). Alternatively, people may compensate for meaning violations in a manner wholly unrelated to the initial source of the violation, by, for example, increasing their commitment to unrelated personal values. This latter process, termed fluid compensation (Allport, 1943), has received much attention and is the basis of several integrative models that now see the pursuit of committed values as a palliative effort to subdue the negative arousal caused by meaning violations (McGregor et al., 2010; Proulx et al., 2012).

### PALLIATIVE COMPENSATION

What is palliative about the pursuit of committed values? As we have discussed, the initial response to threat is the activation of the behavioral inhibition system that increases vigilance, arousal, and avoidance. Behavior is halted and the environment is scanned for an opportunity to either escape from the threat or address the threat directly. Instead of behavioral inhibition, the person under threat would prefer a state of behavioral activation, which will ensue once an opportunity to act has been detected. Such action can be directly aimed at resolving the threat (domain-specific compensation), but action can also involve indirect, relatively abstract goals and values (domain-general compensation). In other words, BIS must be turned into BAS. The defining characteristic of BAS is the approach of a new goal, be it a change in attitude or the affirmation of abstract ideals.

More recent research has demonstrated that the response to meaning violations may indeed result in an increased approach motivation. McGregor et al. (2010) have shown that in response to uncertainty about academic aptitude, students show a rightward error bias in the line-bisection task, which indicates increased left cerebral hemisphericity. Increased activation in the left hemisphere is in turn associated with the motivation to approach (Drake and Myers, 2006; Nash et al., 2010), as described earlier. In a second study, they showed that students also associated their own self more with an approach motivation after the uncertainty manipulation, as measured through an adapted implicit association test (Greenwald et al., 1998), especially if the students’ ideals have been made salient (McGregor et al., 2012).

Research on the predicted positive affect associated with the motivation to approach has so far not been thoroughly investigated. Existing research is mostly limited to correlational work that does not fully disentangle positive affect caused by the positive associations in the environment (e.g., the presence of food or an attractive person) or the actual approach oriented mindset. Nonetheless, many studies do show there is a link. Anhedonia—a diminished capacity to experience pleasure—has been associated with a decreased approach motivation, and could even serve as a better measure of hedonic deficit than commonly used measures of anhedonia (Germans and Kring, 2000). More generally, approach motivation has been linked to well-being (see, Elliot, 2008, chap. 24) and many models link approach to positive emotional states such as excitement and elation, whereas an avoidance motivation is linked to anxiety and fear (Carver, 2004).

Additional evidence for the positivity associated with approach comes from research comparing a personal goal either in approach-oriented terms or avoidance-oriented terms. An approach-oriented goal (e.g., “I will try to be more entertaining at parties”) versus an avoidance-oriented goal (e.g., “I will try not to be such a bore at parties”) leads to greater reports of subjective well-being. These results have been found for a variety of types of goals, ranging from general goals to specific life goals such as academic and social pursuits (Elliot and Sheldon, 1997; Elliot et al., 2006). Furthermore, it has been shown that neural correlates of well-being indicate a link to approach motivation. Greater left vs. right superior frontal activation has been associated with hedonic well-being and positive affect (Urry et al., 2004). More direct evidence for this contention can be found in a study by Nash et al. (2012). They used EEG to measure approach-related frontal asymmetry and subsequently measured ERN as a result of errors during a Stroop task and a multi-source interference task. In both tasks they found that a higher leftward frontal EEG asymmetry predicted a reduced ERN amplitude. A higher rightward frontal asymmetry predicted the opposite, an increased ERN amplitude. This BIS marker is therefore affected by motivational orientation in such a way that approach seems to reduce the experience of conflict. Although more evidence is required, there is support for the contention that the motivation to approach is associated with positive affect and could serve as an effective comforting strategy.

## INDIVIDUAL DIFFERENCES IN PALLIATIVE COMPENSATION

We have thus far reviewed evidence for the proposition that meaning violations induce a state of anxiety and inhibition, which in turn must be overcome by approach-oriented behavior. We now address the extent to which this process is impacted by individual difference factors, with specific emphasis on the BPSM of threat and challenge (Blascovich and Tomaka, 1996). The BPSM of arousal distinguishes between physiological states associated with threat and challenge. Challenge results when an individual evaluates one’s own resources as meeting the demands of the situation. Threat is the result of demands that we (subjectively) determine cannot be met. This distinction is often discussed as an either/or reaction, in that a situation is either perceived as challenging or threatening. However, this model can be linked to the response to meaning violation findings we have reviewed here. Instead of a meaning violation being immediately categorized as something that can be overcome, we argue that meaning violations (e.g., experiences of mortality reminders, behavioral dissonance, or perceptual errors) are responded to as initially ‘threatening,’ that is, affecting our appraisal of the situation as a conflict that potentially exceeds our demands. After this initial response, various factors influence whether the meaning violation is dealt with, or in BPSM terminology, is seen as a challenge that can be met. Support for this integration is not new and initial steps have already been made by Blascovich (2008) himself. He has argued that threat can be mapped onto behavioral inhibition avoidance and challenge onto behavioral approach. The question becomes: which factors influence the transition from threat to challenge?

### SELF-ESTEEM

One such factor is self-esteem. Self-esteem can be considered a trait that determines the extent to which one feels they possess the resources necessary to cope with obstacles and attain goals. High self-esteem should make one feel capable of dealing with obstacles, which are therefore experienced as more challenging and less threatening, facilitating the switch to a behavioral approach state. High levels of trait self-esteem are linked to behavioral approach (Baumeister et al., 1989; Heimpel et al., 2006) and it has also been shown that people with high self-esteem favor approach-oriented goals over avoidance-oriented goals (Tice, 1991; Cavallo et al., 2009). With low self-esteem, the transition to approach might take longer, or fail to occur at all.

In general, self-esteem is related to positive outcomes in life (Taylor et al., 2003a,b; Swann et al., 2007), but self-esteem has also been specifically linked to increased defensiveness against meaning violations. In response to mortality reminders, for example, people with high levels of self-esteem do not show the typical defensive behavior seen in response to these violations (Pyszczynski et al., 2004). For low self-esteem people, however, we observe the opposite. They appear more cautious and inhibited following meaning violations (Vohs and Heatherton, 2001; Cavallo et al., 2009; McGregor et al., 2009), and it appears as though they reside longer in the BIS state than people with high self-esteem. This has negative consequences for well-being, and could even result in serious psychological disorders, as prolonged exposure to anxious arousal can lead to depression and PTSD (Routledge et al., 2010; Pyszczynski and Kesebir, 2011).

### NEUROTICISM

A second important factor that influences the transition from BIS to BAS is the personality trait neuroticism, such that many of the responses to meaning violations are enhanced for those high in neuroticism. Neurotic people are more likely to interpret evocative cues as a violation. For example, they find reminders of sex a greater violation of meaning because it possibly reminds them of their mortality (Goldenberg et al., 1999) and they respond more strongly when their mortality is made more salient explicitly (Arndt and Solomon, 2003). Physiologically, they respond with increased severity to experiences that arouse uncertainty by demonstrating a higher negativity response after receiving no feedback about how they performed, as compared to receiving positive or negative feedback about their performance (Hirsh and Inzlicht, 2008). In fact, more than half a century ago, Eysenck (1951) already proposed that neuroticism is linked to general cortical arousability.

Although the conceptualization of a general physiological arousal is too vague and likely inaccurate, research has accumulated that demonstrates reliable biological correlates to neuroticism (Canli, 2004; DePascalis, 2004). Several theories suggest that neuroticism is the result of an especially sensitive neural comparator, a mechanism that detects mismatches between actual and expected states of the world (Carver and Scheier, 1990; Eisenberger et al., 2005). As discussed in an earlier section, the ACC is responsible for the detection of violated expectations or conflicts in general. People high in neuroticism should therefore show increased activity in the ACC in response to discrepancies; a prediction supported by the findings of Eisenberger et al. (2005). They found that activity in the ACC during a discrepancy detection task was positively correlated with self-reported neuroticism. In line with the use of the BPSM in this review, neuroticism has been linked to threat appraisals of stressors, as opposed to challenge appraisals (Schneider, 2004). As a result, they will show prolonged BIS activation and could benefit from strategies aimed at adopting an approach orientation.

### VALUE AND GOAL COMMITMENT

A final example of an individual difference factor that is relevant to dealing with meaning violations is the extent to which one is committed to readily activated values and goals. Fluid compensation processes imply that as long as a given meaning violation does not require an immediate response, there is always the possibility of pursuing more abstract and situation-independent goals, such as reaffirming one’s ideals and establishing new goal pursuits. Having these values and goals readily available might affect the appraisal of violations in terms of challenge. Support for this idea can be found in an experiment performed by Inzlicht and Tullett (2010), who primed participants with religion or let participants affirm their religious convictions. Interestingly, this led to reduced ERN activity (i.e., reduced BIS activation), but only for committed believers. The presence of a readily available value to pursue can be interpreted as having the resources to deal with the meaning violation—to feel challenged instead of threatened. Similarly, this effect on ERN activity has been found for trait levels of religious zeal and belief in God (Inzlicht et al., 2009). Additionally, the affirmation of personal values buffers neuroendocrine and psychological stress responses, especially so among people with high self-esteem (Creswell et al., 2005). Adopting meaningful ideologies, values, or worldviews could therefore be an important step in not just living a philosophically satisfying life, but also defending oneself against various meaning violations.

## FUTURE DIRECTIONS

It is clear that much progress has been made in the threat-compensation literature in determining how people respond to various meaning violations. However, certain areas remain relatively underinvestigated.

Research on the palliative function of approach motivation is limited. Although it has been shown that approach motivation leads to reduced signs of BIS activation (Nash et al., 2012), this has only been shown in the case where approach is measured before a meaning violation. Nash et al. (2012) measured baseline levels of approach-related left frontal EEG activity and found that this predicted a reduced ERN amplitude in response to conflicts in a task that followed. Ideally, we would also observe physiological markers of approach following meaning violations. Current research has thus far only demonstrated indirect measures of approach motivation, for example through self-report, implicit measures of approach, or the line-bisection task (McGregor et al., 2010). Direct measures of BAS activation have yet to be investigated.

The findings we have presented here mostly relate meaning violations to only a few possible neural substrates of BIS and BAS activation. However, BIS and BAS are complex psychological states that involve many different brain areas. These include structures related to regulatory functions such as the frontal areas (e.g., dorsolateral prefrontal cortex, inferior frontal regions), but also areas related to stress such as the amygdala, insula, substantia nigra and bed nucleus of the stria terminalis complex (Schlund et al., 2011, 2013). Although these structures could undoubtedly enhance our understanding of how people respond to meaning violations, the threat-compensation literature has yet to research the link between meaning violations and these structures more concretely.

Most importantly, however, the literature is in need of experimental designs in which the full process, from violation to approach, is tested. These experiments would involve participants being presented with a meaning violation: a reminder of mortality, goal uncertainty, the loss of control, perceptual anomalies, or cognitive conflict, more generally. This should result in direct activation of the BIS as reflected by activity in the ACC or related neutral structures and peripheral measures of arousal such as cardiac activity or skin conductance. After a delay, or when an opportunity is presented to affirm one’s personal values, the motivation to approach should be made visible, through measures such as the line bisection task (indirectly) or neural activity in the left prefrontal lobe (directly). Following this approach state, measures of BIS should show reduced activity, thereby confirming the palliative functioning of approach. So far no studies have been reported that fully present this process.

Further research might also focus on practical applications of these findings. The research on individual differences in the response to meaning violations have shown that having readily available sources of meaning can help reduce the anxiety in response to threats. Also, framing goals in an approach-oriented manner is conducive to well-being. These findings could potentially translate to therapeutic settings where greater emphasis is put on having valued sources of meaning in one’s life. These often abstract sources—ideologies, moral systems, worldviews—have the benefit of being relatively easily accessible and their abstract nature might also make them less likely to be thwarted by situational constraints (see, McGregor et al., 2012). Their pursuit can largely go unhindered, therefore serving as an effective coping strategy.

## CONCLUSION

Meaning violations evoke a specific stress response that begins with a defensive reaction marked by anxiety, vigilance, and avoidance—a state of behavioral inhibition. People respond to this aversive state by approaching their values, ideologies, and worldviews. We suggest, in line with the BPSM of arousal regulation, that all meaning violations initially cause an inhibitory threat-response that subsequently switches to a state of approach; especially when factors such as self-esteem, personality, and the availability of commitments impact one’s appraisal of the situation. Nevertheless, it is not the content of affirmed values, ideologies, or worldviews that alleviates stress, but rather the state of approach—in and of itself—that people find comforting. This integration of findings across the threat-compensation literature is but one among many in a recent surge of integrative efforts in this field (e.g., Jonas et al., 2014). We expect these developments will provide new insight into the this literature, as well as well related fields of research.

## Conflict of Interest Statement

The authors declare that the research was conducted in the absence of any commercial or financial relationships that could be construed as a potential conflict of interest.
